# Durability and inflammogenic impact of carbon nanotubes compared with asbestos fibres

**DOI:** 10.1186/1743-8977-8-15

**Published:** 2011-05-13

**Authors:** Megan J Osmond-McLeod, Craig A Poland, Fiona Murphy, Lynne Waddington, Howard Morris, Stephen C Hawkins, Steve Clark, Rob Aitken, Maxine J McCall, Ken Donaldson

**Affiliations:** 1 CSIRO Food and Nutritional Sciences, 11 Julius Avenue, North Ryde NSW 2113, Australia; 2 MRC/University of Edinburgh Centre for Inflammation Research, ELEGI Colt Laboratory, Queens Medical Research Institute, 47 Little France Crescent, Edinburgh EH16 4TJ, UK; 3 CSIRO Materials Science and Engineering, 343 Royal Parade, Parkville VIC 3052, Australia; 4 Safe Work Australia, GPO Box 681, Canberra ACT 2601, Australia; 5 CSIRO Materials Science and Engineering, Bayview Avenue, Clayton VIC 3168, Australia; 6 Institute of Occupational Medicine, Research Avenue North, Riccarton Edinburgh E14 4AP, UK

## Abstract

**Background:**

It has been suggested that carbon nanotubes might conform to the fibre pathogenicity paradigm that explains the toxicities of asbestos and other fibres on a continuum based on length, aspect ratio and biopersistence. Some types of carbon nanotubes satisfy the first two aspects of the fibre paradigm but only recently has their biopersistence begun to be investigated. Biopersistence is complex and requires *in vivo *testing and analysis. However durability, the chemical mimicking of the process of fibre dissolution using *in vitro *treatment, is closely related to biopersistence and more readily determined. Here, we describe an experimental process to determine the durability of four types of carbon nanotubes in simulated biological fluid (Gambles solution), and their subsequent pathogenicity *in vivo *using a mouse model sensitive to inflammogenic effects of fibres. The *in vitro *and *in vivo *results were compared with well-characterised glass wool and asbestos fibre controls.

**Results:**

After incubation for up to 24 weeks in Gambles solution, our control fibres were recovered at percentages consistent with their known *in vitro *durabilities and/or *in vivo *persistence, and three out of the four types of carbon nanotubes tested (single-walled (CNT_SW_) and multi-walled (CNT_TANG2_, CNT_SPIN_)) showed no, or minimal, loss of mass or change in fibre length or morphology when examined by electron microscopy. However, the fourth type [multi-walled (CNT_LONG1_)] lost 30% of its original mass within the first three weeks of incubation, after which there was no further loss. Electron microscopy of CNT_LONG1 _samples incubated for 10 weeks confirmed that the proportion of long fibres had decreased compared to samples briefly exposed to the Gambles solution. This loss of mass and fibre shortening was accompanied by a loss of pathogenicity when injected into the peritoneal cavities of C57Bl/6 mice compared to fibres incubated briefly. CNT_SW _did not elicit an inflammogenic effect in the peritoneal cavity assay used here.

**Conclusions:**

These results support the view that carbon nanotubes are generally durable but may be subject to bio-modification in a sample-specific manner. They also suggest that pristine carbon nanotubes, either individually or in rope-like aggregates of sufficient length and aspect ratio, can induce asbestos-like responses in mice, but that the effect may be mitigated for certain types that are less durable in biological systems. Results indicate that durable carbon nanotubes that are either short or form tightly bundled aggregates with no isolated long fibres are less inflammogenic in fibre-specific assays.

## Background

**Figure 1:**
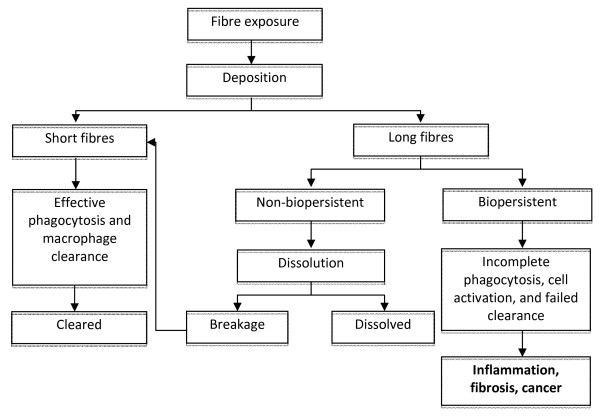
**Paradigm for the role of length and biopersistence in the pathogenic effects of fibres**. Schematic outlining the possible fates for fibres *in vivo *based on length and biopersistence (from Donaldson *et al. *2006). Long, narrow (not depicted) and biopersistent fibres that deposit in the distal lung regions may cause inflammation, fibrosis and/or cancer.

It has been suggested that the potential pathogenicity of carbon nanotubes (CNTs) might conform to the 'fibre pathogenicity paradigm', by which a fibre's pathogenicity can be predicted on a continuum based on its length and biopersistence (Figure 1), as well as aspect ratio. On this continuum, fibres that are more likely to induce 'asbestos-like' pathologies such as asbestosis, lung cancer, and mesothelioma will be narrow enough that they can reach the distal lung upon inhalation, long enough to be incompletely engulfed by macrophages during clearance, and resistant to chemical attack or breakage in a biological environment. Deposition of long, narrow, biopersistent fibres into the distal lung region can be problematic to health if local macrophage-mediated clearance is only partially successful and instead results in a state of chronic macrophage stimulation (so-called 'frustrated phagocytosis'). The sustained release of inflammatory mediators may then lead to inflammatory, fibrotic and carcinogenic outcomes [1]. Oberdorster (2000) regards fibres > 20 μm long as being of sufficient length to induce these kinds of fibre-induced pathogenic effects [2].

CNTs possess properties such as exceptional strength, lightness and conductivity that make them valuable for industrial and medical applications [3,4], but can be manufactured to reach potentially pathogenic lengths. Indeed, their morphological similarity to asbestos fibres raised early concerns about possible adverse effects on human health [5]. However CNTs are prone to form aggregates due to strong hydrophobic forces and this could impact on their ability to become airborne as individual fibres at high concentrations under controlled, small-scale conditions [6-8]. Notwithstanding their propensity to aggregate, if individual CNTs or small bundles were to be incidentally or accidentally aerosolised with a respirable aerodynamic diameter it is possible they could align lengthwise with the airstream and reach the distal lung [9]. Additionally, the increased risk of exposure that might arise in large-scale manufacturing environments has been noted [10].

In addition to length and aspect ratio, biopersistence (i.e. the ability of a material to persist in the body in spite of physiological clearance mechanisms) is regarded as one of the most important determinants of a fibre's pathogenicity [2,9]. Biopersistent fibres resist the leaching or solubilisation of structural elements within a biological environment such as the lung-lining fluid or the internal environment of macrophages. Less biopersistent fibres, in contrast, can weaken and break at the weakened points, thus becoming short enough for successful clearance [1]. CNTs were initially thought to be resistant to chemical attack due to their essentially graphitic nature [9], and indeed both single-walled (SW) and multi-walled (MW) CNTs have been shown to persist *in vivo *up to months post-exposure in mice and rats [11-13]. However, other reports have suggested that certain types of SWCNTs and MWCNTs may be subject to degradation in biological environments [14-17]. This broaches the possibility that whilst some CNTs may have pathogenic potential in their pristine form, they may lose this if they are vulnerable to degradation in the biological milieu.

Durability is a key factor in determining fibre biopersistence. Thus, measurement of durability has been used with success to predict biopersistence [1], although it is not without its drawbacks [2]. Here, we describe experiments designed to assess the durability in simulated biological fluid (Gambles solution) of four types of CNTs compared with one type of glass wool fibre (X607) and two types of asbestos fibres [long fibre amosite (LFA) and long fibre chrysotile (LFC)]. The four types of CNTs tested included one single-walled (CNT_SW_) and three multi-walled CNTs ['spinnable' (CNT_SPIN_); 'long' (CNT_LONG1_); and 'tangled' (CNT_TANG2_)], where the names for the latter two samples are taken from Poland *et al. *[18] (Table 1). Following incubation in Gambles solution we assessed the loss of mass as a measure of durability, and then examined a subset of these fibres (following either minimal or lengthy exposure to Gambles solution) for their ability to induce an inflammatory response when injected into the peritoneal cavities of female C57Bl/6 mice in order to assess the impact of durability on CNT fibre-induced pathogenicity. These data should assist in the placement of CNTs on the spectrum of potentially pathogenic fibres in order to inform their safe use.

**Table 1:** Fibres investigated in this study and rationale for their use

Fibre	Source	Rationale for Use
**X607**	Rockwool International	Non-durable glass wool control

**LFA**	Archival samples	Durable amosite asbestos control

**LFC**	Archival samples	Non-durable chrysotile asbestos control

**CNT_SW_**	Sigma-Aldrich	Unknown durability

**CNT_SPIN_**	CSIRO Australia	Unknown durability

**CNT_LONG1_**	Mitsui & Co	Unknown durability

**CNT_TANG2_**	NanoLab Inc	Unknown durability

## Results

### *In vitro *durability of test samples

**Figure 2:**
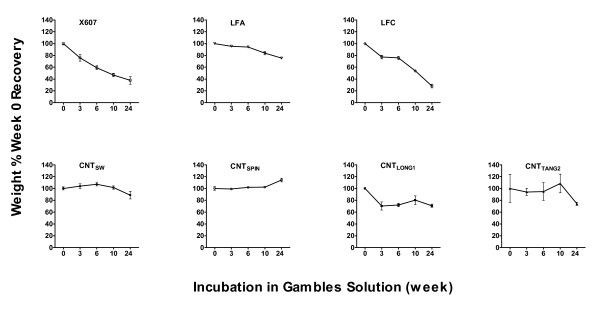
**Durabilities of CNT samples and controls in simulated biological fluid**. The weights of quadruplicate samples of glass wool fibre (X607), asbestos fibres (LFA, LFC) and CNT test samples (CNT_SW_, CNT_SPIN_, CNT_LONG1 _and CNT_TANG2_), recovered from Gambles solution following incubation for various times are expressed relative to the weight of sample recovered at 0 weeks.

Gambles solution is a balanced electrolyte solution similar to the electrolyte environment of biological systems. Its pH is adjusted to mimic that inside macrophage phagolysosomes, potentially the most degradative environment that a particle should encounter following lung deposition and macrophage uptake. Fibre durability was assessed by incubating controls and test samples in quadruplicate in Gambles solution for defined times throughout a 24 week experimental period, and determining percent recoveries of original weight after filtering and drying (Figure 2). Samples recovered at 100% of their original weights are described as 100% durable.

Of the control fibres, the non-durable glass fibre, X607, was recovered with ~40% of its original weight after the 24 week incubation, while the "durable" asbestos fibre, LFA, was recovered at ~75%. The other asbestos fibre examined here, LFC, was recovered with ~30% of original weight over the same period. None of the CNT samples showed a significant loss of mass by week 24 with the exception of CNT_LONG1_, which was recovered at only ~70% of its original weight at all time-points from week 3 onward. A small but statistically significant increase in mass was observed for the CNT_SPIN _sample at week 24. CNT_SW _and CNT_TANG2 _showed some variation in percent recoveries across the time-points but these were generally neither consistent nor statistically significant (Table 2).

**Table 2:** Weights of samples recovered from Gambles solution at 0 weeks and various incubation times to 24 weeks

Sample ID	Incubation Period (week)	% Week 0 Recovery ± SE
**X607**	0	100 ± 2.09
	
	3	75.8 ± 5.46 *
	
	6	59.2 ± 3.2 *
	
	10	46.86 ± 3.07 *
	
	24	37.82 ± 6.6 *

**LFA**	0	100 ± 1.55
	
	3	95.53 ± 1.48
	
	6	94.13 ± 1.2
	
	10	83.76 ± 2.62 *
	
	24	75.43 ± 1.33 *

**LFC**	0	100 ± 0.3
	
	3	77.26 ± 2.93 *
	
	6	75.8 ± 2.6 *
	
	10	53.79 ± 1.42 *
	
	24	28.23 ± 3.03 *

**CNT_SW_**	0	100 ± 2.46
	
	3	103.92 ± 3.94
	
	6	107.08 ± 2.85
	
	10	101.51 ± 2.69
	
	24	88.68 ± 6.4

**CNT_SPIN_**	0	100 ± 3
	
	3	99.14 ± 1.23
	
	6	101.92 ± 1.39
	
	10	102.18 ± 1.36
	
	24	114.18 ± 2.91*

**CNT_LONG1_**	0	100 ± 1.36
	
	3	70.37 ± 6.75 *
	
	6	71.99 ± 3 *
	
	10	80.19 ± 7.41
	
	24	70.76 ± 2.59 *

**CNT_TANG2_**	0	100 ± 23.85
	
	3	94.23 ± 6.01
	
	6	94.84 ± 14.94
	
	10	108.39 ± 15.83
	
	24	74.06 ± 2.58

Recovered weights of incubated samples in quadruplicate are expressed as percent relative to the recovered weight at 0 weeks, and presented here as mean ± standard error (SE) of the mean. The statistical significance of differences from the recoveries at 0 weeks were assessed by one-way ANOVA with Tukey's Multiple Comparison post-test. A statistically significant difference from the recovery at 0 weeks was set at *p *< 0.05 and is denoted by an asterisk.

Sources of error in sample recoveries may include sample preparation by different operators, loss of sample during refreshing of Gambles solution, and loss of sample during filtration, reflecting general difficulties in handling CNT samples. We estimate that these sources of error may account for up to 20% of variation in sample recovery. Therefore, variation of 20% from the original mass may not reflect true differences in recoveries, unless the 20% was part of a consistent trend across all time-points. On this basis, despite reaching apparent statistical significance for one measurement, we suggest that the variations in percent recoveries for CNT_SW_, CNT_TANG2 _and CNT_SPIN _reflect experimental error, as consistent trends were not evident across all time-points for these samples. In contrast, the loss of mass observed for CNT_LONG1 _was consistent and statistically significant across most time-points. Of the control fibres, we suggest that percent recoveries for X607 and LFC reflect true mass loss, whereas the small mass loss for LFA over the 24 week period may be due to the loss of small fibres in the sample (discussed later).

**Figure 3:**
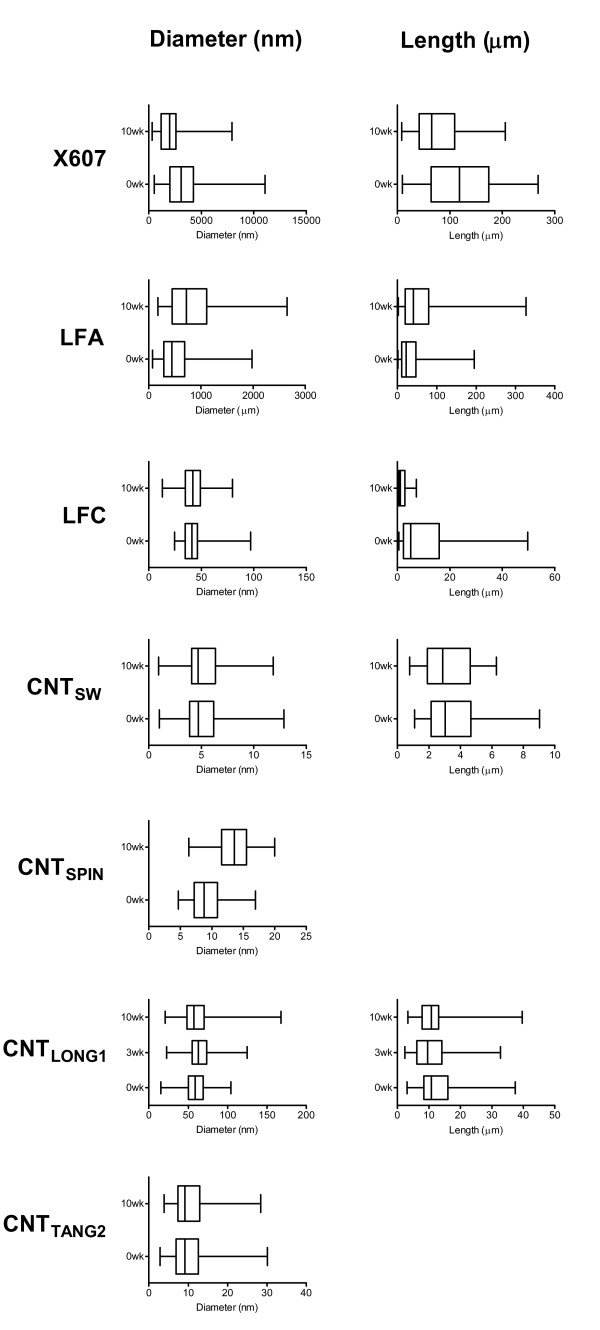
**Effect of incubation in Gambles solution on fibre widths and lengths**. Boxplots showing the distribution of fibre widths and lengths (nm) in samples that had been incubated in Gambles solution for 0 weeks or 10 weeks. The line in the box represents the median value of measurements from TEM images, and the edges of the box represent the lower and upper quartiles. The ends of the whiskers represent minimum and maximum values.

**Figure 4:**
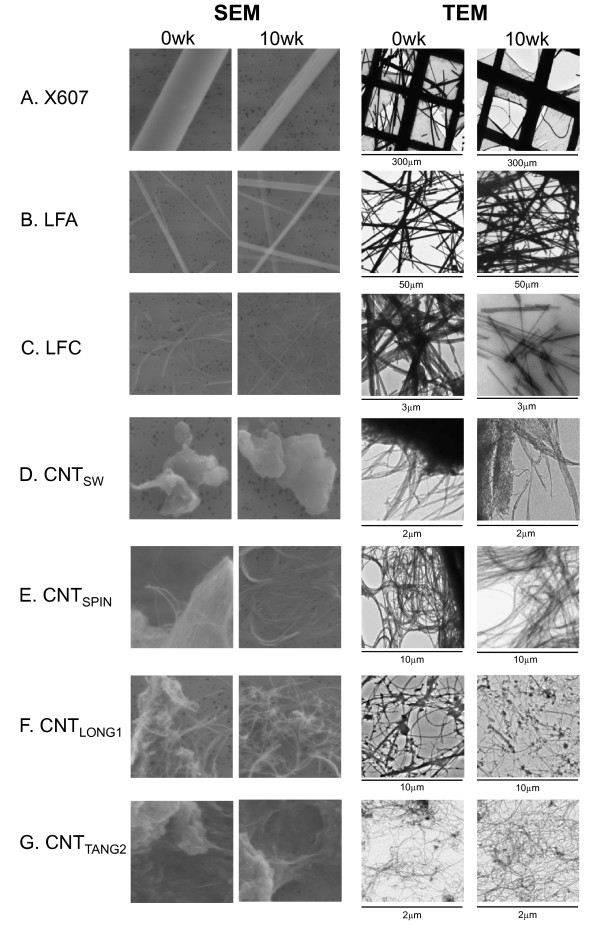
**Appearance of fibres before and after incubation in Gambles solution**. Representative SEM images of samples after 0 weeks or 10 weeks incubation in Gambles solution are shown at 5.0 K magnification in the two panels on the left. TEM images of equivalent samples, at indicated magnifications, are on the right.

TEM images of samples that had been incubated for 0 weeks or 10 weeks in Gambles solution were taken at various magnifications, and the widths and lengths of fibres in these images were measured using Image J (NIH) calibrated software (Table 3). Size distributions are shown in Figure 3, and representative SEM and TEM images in Figure 4.

**Table 3:** Average widths and lengths, and length distributions, of samples incubated in Gambles solution for 0 weeks or 10 weeks, as determined by TEM measurements

Sample	Incubation Period (week)	Width (nm) ± SE	Length μ(m) ± SE	% ≥ 100 μm	100 μm > % ≥ 20 μm	20 μm > % ≥ 15 μm	15 μm > % ≥ 10 μm	10 μm > % ≥ 5 μm	% < 5 μm
**X607**	0	3500 ± 190	123 ± 7	58	36	4	1	1	0
	
	10	2100 ± 169*	76 ± 5*	33	55	6	3	2	0

**LFA**	0	550 ± 39	34 ± 3	5	50	8	16	13	8
	
	10	820 ± 52*	56 ± 4*	18	56	6	6	7	6

**LFC**	0	42 ± 1	10.8 ± 1	0	20	6	9	17	49
	
	10	43 ± 2	1.9 ± 0.3*	0	0	0	0	0	100

**CNT_SW_**	0	5 ± 0.3	3.6 ± 0.2	0	0	0	0	19	81
	
	10	5 ± 0.3	3.2 ± 0.4	0	0	0	0	18	82

**CNT_SPIN_**	0	9 ± 0.3	NAs	NAs	NAs	NAs	NAs	NAs	NAs
	
	10	14 ± 0.3*	NAs	NAs	NAs	NAs	NAs	NAs	NAs

**CNT_LONG1_**	0	60 ± 2	12.4 ± 0.5	0	10	20	29	37	4
	
	3	65 ± 2	10.9 ± 0.5*	0	8	10	27	45	10
	
	10	63 ± 2	11.1 ± 0.4*	0	4	9	42	38	7

**CNT_TANG2_**	0	10 ± 0.4	NAs	NAs	NAs	NAs	NAs	NAs	NAs
	
	10	10 ± 0.4	NAs	NAs	NAs	NAs	NAs	NAs	NAs

The dimensions of fibres, for which a start and end point could be identified in representative TEM images, were measured using Image J (NIH) calibrated software. The mean widths and lengths of samples incubated for 10 weeks were compared to their equivalent 0 weeks samples by an unpaired t-test, where statistical significance was set at *p *< 0.05 (denoted by an asterisk). Fibre dimensions were additionally determined for CNT_LONG1 _at 3 weeks incubation to assess whether the shortening seen at 10 weeks was part of a consistent trend.

NAs: not assessable

**Note**: Due to the difficulty in identifying and measuring individual CNTs within bundles in TEM images, these numbers should not be taken as absolute but indicative of trends only.

The X607 sample (Figure 4A) contained the largest fibres assessed here, with widths in the micrometre range and average lengths markedly greater than 20 μm. Whilst fewer long fibres remained after 10 weeks incubation, of those that remained a high proportion were still very large (Table 3, Figure 3). Nevertheless, the average width of these remaining fibres decreased from 3.5 to 2.1 μm and length decreased from 123 μm to 76 μm.

LFA showed an apparent increase in average fibre width and length as a result of the incubation. The proportion of long fibres also appeared to increase (Table 3, Figure 3). It is possible that the loss of mass at the last two time-points for this sample (Figure 2) reflects the loss of smaller fibres, leaving, on average, a greater proportion of larger fibres remaining in the recovered 75%. This would potentially bias the average width and length to the larger end of the size distributions. The fibres remaining at 10 weeks did not show any morphological differences from those at 0 weeks when viewed by electron microscopy (Figure 4B).

LFC showed no difference in average fibre width with incubation but did show a marked decrease in length (Table 3, Figure 3). We note that at 0 weeks the LFC sample comprised a mixture of fibrils and ropes of fibrils (Figure 4C). All were measured in the 0 weeks sample, potentially assigning erroneously large measurements to individual fibres, whereas at 10 weeks only small fibrils remained, resulting in average length measurements at the shorter end of the distribution. Given this sample also showed a marked loss of mass, it is probable that the measured loss of length accurately reflects fibre shortening in addition to the breaking up of large fibre bundles.

CNT_SW _did not show an alteration in average fibre width or length arising from incubation in Gambles solution (Table 3, Figure 3). It also showed little morphological change under electron microscopy (Figure 4D), with the majority of fibres forming large clumps. Generally, individual fibres could be seen only at the edges.

Lengths for CNT_SPIN _could not be determined due to their very long, hair-like nature, making the starts and ends of individual tubes virtually impossible to identify (Figure 4E). The average width showed an apparent slight increase after 10 weeks incubation in Gambles solution (Table 3, Figure 3). We cannot explain this result, apart from noting the difficulty in identifying individual fine tubes in this sample.

CNT_LONG1 _samples that had been incubated in Gambles solution for 3 weeks and 10 weeks showed no decrease in average fibre width compared with the starting material, but did show small, statistically significant decreases in the average length. Additionally, there was a decrease in the proportion of long fibres present compared to samples incubated for 0 weeks, with approximately 50% fewer fibres with lengths >15 μm in samples incubated for 10 weeks compared to 0 weeks (Table 3, Figure 3). TEM images at 0 weeks and 10 weeks show that many fibres in this sample contained what may be residual catalyst material or amorphous carbon (Figure 4F), as well as a large number of what appear to be curled CNTs decorating the straighter fibres (more clearly seen in images at higher magnification, not shown).

The lengths of CNT_TANG2 _fibres were not able to be determined due to their 'tangled' nature and subsequent difficulties in identifying discrete tubes from start to end. Their average width (Table 3, Figure 3) and general morphology (Figure 4G) did not change as a result of the incubation in Gambles solution when viewed by electron microscopy.

### *In vitro *determination of the impact of 1 h bath sonication on mass loss and changes in fibre dimensions for CNT_LONG1_

It has been reported elsewhere that sonication can mechanically shear CNTs, with the degree of damage depending on the type of CNT and sonication conditions [19]. As our 0 weeks samples were not sonicated, but the other samples were, we performed a small supplementary experiment to determine if the 1 h gentle bath sonication of the incubated fibres was responsible for the fibre shortening observed for the CNT_LONG1 _sample, rather than the incubation in Gambles solution. In this experiment, CNT_LONG1 _samples were either first sonicated for 1 h in Gambles solution, or were simply added to Gambles solution and then immediately filtered, dried and weighed, and then fibre lengths were measured, replicating the conditions by which we produced our original 0 weeks samples. The results showed no mass loss resulting from the 1 h sonication, and no statistical difference in the average fibre length or proportion of long fibres (data not shown). In addition, we examined filtrates from the original 0 weeks and 10 weeks samples, as well as from the new 0 h and 1 h sonicated samples. Only the original samples incubated for 10 weeks in Gambles solution contained CNT debris (CNT fragments approximately 500 nm long). These results provide strong evidence for the observed mass loss and fibre shortening of 10 weeks CNT_LONG1 _samples being caused by incubation in Gambles solution and not by sonication under our experimental conditions.

### *In vivo *inflammogenic response to test samples

**Figure 5:**
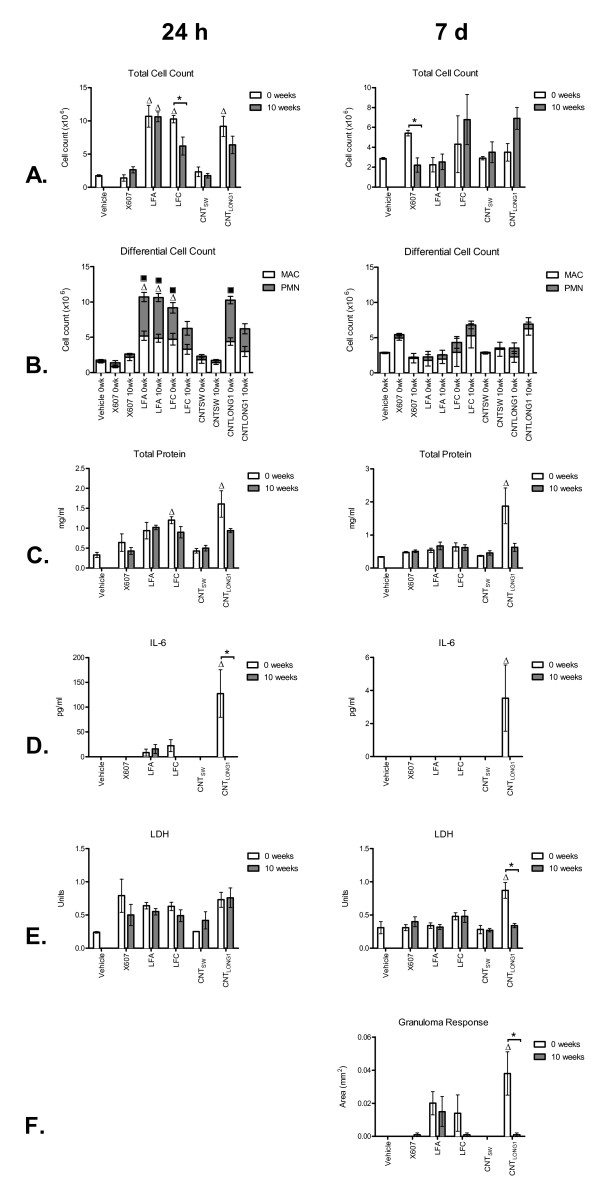
**Inflammatory responses to test samples injected into the peritoneal cavities of mice**. Samples of vehicle only, X607, LFA, LFC, CNT_SW _and CNT_LONG1_, incubated for either 0 weeks or 10 weeks in Gambles solution, were injected into the peritoneal cavities of mice, and inflammatory responses were assessed at 24 h (panels on left) and 7 d (panels on right) post-injection. The inflammatory response was assayed by total (A) and differential (B) cell counts to identify infiltration of immune cells into the peritoneal cavity, total protein (C) to indicate increased permeability in the peritoneal cavity, IL-6 (D) as a measure of inflammatory cytokines, LDH (E) to indicate damage to cellular membranes, and the development of fibrotic plaques at 7 d (G). Note that the scales of the vertical axes vary. Differences between mice treated with the same fibre samples incubated for 0 weeks or 10 weeks were assessed by unpaired t-tests, with statistical significance set at *p *< 0.05 (denoted by an asterisk). Differences between mice treated with fibres that had been incubated for 0 weeks or 10 weeks compared to mice treated with vehicle only were assessed by one-way ANOVA with Tukey's Multiple Comparison post-test, with statistical significance set at *p *< 0.05 (denoted by an unshaded triangle). For the differential cell counts, statistically significant differences between the numbers of macrophages (MAC) and polymorphonuclear leukocytes (PMNs) in treated mice compared to control mice are denoted by an unshaded triangle or a black square, respectively.

Samples that had been incubated in Gambles solution for 0 weeks or 10 weeks were filtered and resuspended in 0.5% bovine serum albumin (BSA):saline at a presumed 100% recovery of mass, and a presumed mass of 50 μg was injected into the peritoneal cavities of female C57Bl/6 mice. Mice were sacrificed at 24 h or 7 d post-injection and the peritoneal cavities were washed and lavage fluid collected. To identify the presence of an acute inflammatory response, a number of assays were performed: total and differential cell counting to identify possible infiltration of immune cells into the peritoneal cavities in response to the treatments (Figure 5A, B); measurement of total protein as a marker for increased permeability in the peritoneal cavity (Figure 5C); measurement of the cytokine, IL-6, as a marker for the release of inflammatory cytokines (Figure 5D); and measurement of LDH as a marker for damage to cellular membranes (Figure 5E). The development of fibrotic plaques at 7 d was also assessed (Figure 5F). CNT_TANG2 _and CNT_SPIN _were not included in the *in vivo *analysis because the former had been shown previously to be non-pathogenic in a similar study [18], and the latter was not able to be dispersed well enough to inject a reliable amount of sample into the mice.

**Figure 6:**
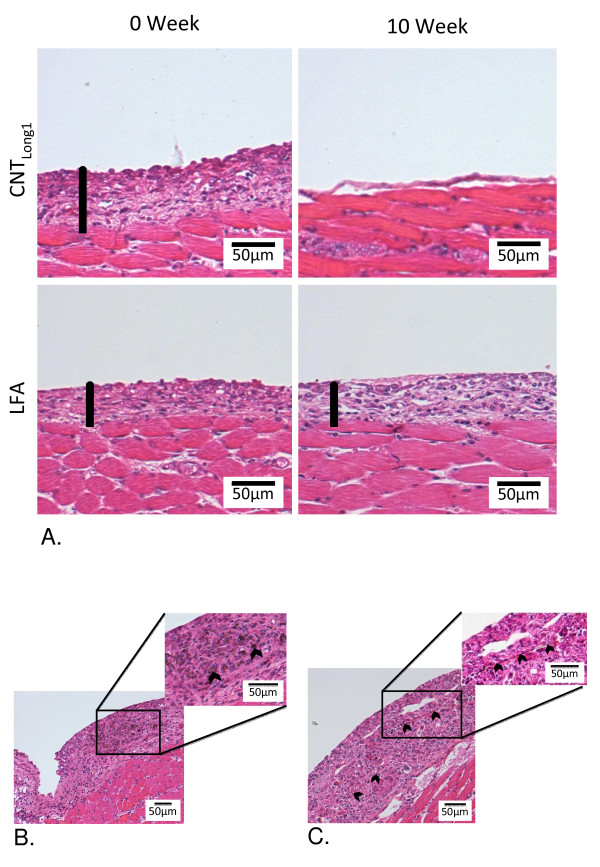
**Fibrotic plaques on the peritoneal face of the diaphragm following intraperitoneal instillation of CNT_LONG1 _and LFA fibres**. A. Differential development of fibrotic plaques on the peritoneal face of the diaphragm following the injection of CNT_LONG1 _or LFA fibres that had been incubated for 0 weeks (left panels) or 10 weeks (right panels). Black rods indicate the depth of the fibrotic plaque and show that 10 weeks incubation in Gambles solution did not impact the response to LFA fibres, whereas the fibrotic response from CNT_LONG1 _0 weeks was largely absent in mice injected with CNT_LONG1 _10 weeks. Co-localisation of fibrotic plaques with CNT_LONG1 _fibres (B) or LFA fibres (C) (indicated by black chevrons) is also shown.

X607 did not elicit a statistically significant inflammatory response with the exception of the total cell count for the 7 d sample, regardless of incubation time in Gambles solution, although it should be noted that some biomarkers were elevated compared to levels in mice treated with vehicle only. LFA elicited an acute inflammatory response in mice in addition to the development of fibrotic plaques by 7 d, regardless of incubation time, suggesting that long-term incubation did not alter its pathogenicity. In contrast, LFC that had been incubated in Gambles solution for 0 weeks induced total and differential cell counts, total protein, and IL-6 levels indicative of an acute inflammatory response that subsequently subsided by 7 d alongside the development of a fibrotic response, whereas LFC that had been incubated for 10 weeks had a reduced inflammogenic response at 24 h, although interestingly, not at 7 d; at 7 d the total cell count was higher in mice injected with the LFC incubated for 10 weeks compared to LFC that had been incubated for 0 weeks, as well as higher than LFA. However, LFC that had been incubated for 10 weeks did not induce a fibrotic response at 7 d whereas LFC incubated for 0 weeks or LFA incubated for 0 weeks or 10 weeks did. CNT_SW _did not induce an inflammatory response under the experimental conditions studied here. CNT_LONG1 _incubated in Gambles solution for 0 weeks induced an acute inflammatory response at 24 h post-injection into mice that did not subside by 7 d, and also induced a strong fibrotic response at 7 d. However, CNT_LONG1 _that had been incubated in Gambles solution for 10 weeks was less pathogenic in mice, inducing reduced inflammatory and fibrotic responses compared to 0 weeks (Figure 6).

The inflammatory responses *in vivo*, combined with the durability data *in vitro*, are summarised in Table 4. Taken together, the data indicate that CNT_LONG1 _and LFC showed some mass loss and fibre shortening with long-term incubation in Gambles solution, with a concomitant mitigation of the pathogenicity seen in mice injected with 0 weeks samples. LFA that had been incubated for 10 weeks, on the other hand, also showed a loss of mass comparable to CNT_LONG1 _at the same time-point, but no fibre shortening, and did not lose its pathogenicity (Figure 6). These observations suggest that the loss of pathogenicity under these experimental conditions may have been associated more with the decreased proportion of long fibres than loss of mass.

**Table 4:** Summary of results from experiments to determine durability, and acute and 7 d inflammatory responses *in vivo*, for CNT samples and controls

		Fibre Changes		Acute Inflammatory Response (24 h)					7 d Inflammatory Response					
		**Loss of Mass**	**Fibre Shortening**	**Total Cell**	**PMN Infiltration**	**Total Protein**	**IL-6**	**LDH**	**Total Cell**	**PMN Infiltration**	**Total Protein**	**IL-6**	**LDH**	**Granuloma Response**

**X607**	0 week	N/A	N/A	-	-	-	-	-	-	-	-	-	-	-
	
	10 week	+	+	-	-	-	-	-	-	-	-	-	-	-

**LFA**	0 week	N/A	N/A	+	+	-	-	-	-	-	-	-	-	Yes (ns)
	
	10 week	+	-	+	+	-	-	-	-	-	-	-	-	Yes (ns)

**LFC**	0 week	N/A	N/A	+	+	+	-	-	-	-	-	-	-	Yes (ns)
	
	10 week	+	+	-	-	-	-	-	-	-	-	-	-	-

**CNT_SW_**	0 week	N/A	N/A	-	-	-	-	-	-	-	-	-	-	-
	
	10 week	-	-	-	-	-	-	-	-	-	-	-	-	-

**CNT_LONG1_**	0 week	N/A	N/A	+	+	+	+	-	-	-	+	+	+	Yes (s)
	
	10 week	+*	+	-	-	-	-	-	-	-	-	-	-	-

This table compiles and summarises data presented in Tables 2 and 3, and Figures 1, 2, 3 and 5. A cross indicates a statistically significant difference between treatment and control (for fibre changes, 10 weeks compared with 0 weeks; for inflammatory responses, response to fibre sample compared with vehicle only). A dash indicates no statistically significant difference.

(+ = statistically significant (*p *< 0.05); - = not statistically significant; (ns) = not significant; (s) = significant; N/A: not applicable)

* Note: The mass loss for CNT_LONG1 _10 weeks was just under statistical significance; however the mass losses at 3 weeks, 6 weeks and 24 weeks were significant, and therefore representative statistical significance has been attributed to the 10 week sample here.

## Discussion

Our study demonstrates that the types of carbon nanotubes investigated here conform to the "fibre pathogenicity paradigm". Where CNTs were injected to the abdominal cavities of mice as long discrete fibres or fibre-like structures, as in the case of CNT_LONG1 _(0 weeks incubation in Gambles solution), an inflammatory and fibrotic response was induced. However, where no or relatively few long fibres were present, as in the case of CNT_LONG1 _after 10 weeks incubation in Gambles solution, where the proportion of fibres >15 μm was markedly reduced, or in the case of CNT_SW_, where the fibrous shape of individual tubes was masked by tight bundling, the inflammatory response was minimal. Our results are consistent with a previous study also using the peritoneal cavity as a surrogate model for exposure of the thoracic mesothelium to particles, where a range of MWCNTs or asbestos fibres were directly injected into the abdominal cavities of mice; only those samples containing long, discrete fibres elicited an "asbestos-like" response [18], while fibres that were either short and/or in the form of tight bundles were not pathogenic. Further, the ability of inhaled MWCNT to reach sub-pleural tissues in mice has also been reported [20,21], indicating that MWCNTs can share a characteristic with asbestos that is critical for the development of asbestos-related mesothelioma [22]. Thus we may expect that a fibre-like pathogenicity might be induced by any type of CNT that reaches the sub-pleural tissue if the CNT presents as a discrete long fibre or fibre-like structure, and that shape persists.

As expected, the control fibres also largely conformed to the fibre pathogenicity paradigm. LFA is known to be durable and pathogenic [23]. Although short fibres of LFA disappeared upon incubation in Gambles solution, accompanied by a small loss of mass, the long fibres endured and, when injected into abdominal cavities of mice, LFA induced a strong inflammatory response consistent with the presence of durable, long fibres with high aspect ratios. X607 is an experimental alkaline earth silica wool with high CaO content (personal communication O. Kamstrup, Rockwool International) found to be non-biopersistent in animal studies [24], while chrysotile asbestos (LFC) is considered to be the least biopersistent of the asbestiform minerals and has shorter clearance half-times in inhalation studies [25,26]. Both of these fibres showed a loss of mass upon incubation in Gambles solution, and this was accompanied by surface etching and splitting, and fibre thinning and/or shortening, respectively, as revealed by electron microscopy. LFC injected into mice after 0 weeks incubation induced a strong inflammatory response, whereas the incubation-mediated degradation mitigated its inflammatory potential. Nevertheless, it is interesting to note that at 7 d post-injection, the inflammatory response for LFC was greater than for LFA, but the former did not elicit a fibrotic response whereas the latter did. The development of granulomas is associated with a decrease in the lavageable polymorphonuclear leukocytes and macrophages as the inflammatory response is "walled off" into the granuloma. The switching of inflammatory action to the granulomas means that the leukocytes are less easily lavaged, consistent with our observations for LFC and LFA. In the presence of LFC, it is possible that either the granulomas were slower to develop and did not form within our time-frame, or that ongoing dissolution of the chrysotile fibres in the leukocytes made for fewer granuloma. Indeed, there is evidence that the dissolution of chrysotile can be very rapid *in vivo *[27], and so a very rapid *in vivo *dissolution of LFC here may have elicited a transitory inflammatory response as the magnesium from the chrysotile crystal lattice was released, but no granuloma formed due to the absence of long fibres. The observations for X607 are also interesting. Although X607 showed mass loss and fibre shortening and thinning, nevertheless long fibres with high aspect ratios remained after *in vitro *incubation; but X607 elicited only a minimal inflammatory response in mice regardless of incubation time. This apparent non-conformity to the fibre pathogenicity paradigm could be explained by a comparatively lower *number *of X607 fibres being injected into the mice, as the doses were based on equal mass and the X607 fibres were the thickest and longest. Alternatively, glass inherently may be less inflammogenic in biological systems compared to other types of fibres [28-30], indicating that surface-related properties (such as surface chemistry, surface nanostructure, or curvature) may play a role in the biological response to fibres.

The length and aspect ratio of a fibre over the time of its residence in the lungs will depend on its resistance to degradation. Hence, knowledge of the durability of a CNT sample, together with its ultimate shape, could indicate its pathogenicity if it reaches the pleural cavity. Three of the four types of CNTs investigated here were durable in Gambles solution. These samples, CNT_TANG2_, CNT_SPIN _and CNT_SW_, also revealed minimal changes in morphology following the chemical incubation. In an earlier study, the multi-walled sample, CNT_TANG2_, which exists as tangled, long fibres, was shown to be non-pathogenic when injected in the abdominal cavities of mice [18]. While the difficulties of working with the multi-walled CNT_SPIN _precluded *in vivo *testing here, the single-walled CNT_SW _was injected in the peritoneal cavity of mice and found to be non-inflammatory, regardless of the incubation time. As the mouse peritoneal cavity is responsive to long discrete fibres, but not to compact particles or short fibres, these results are consistent with the shapes of the aggregated CNTs in each sample. Thus, although CNT_TANG2 _and CNT_SW _were durable, the peritoneal cavity was not sensitive to the shape of either [this work and [18]]. It should be noted that the lack of an inflammatory response in the abdominal peritoneal cavity from these CNT_SW _does not necessarily imply they will be benign in the lung (not investigated here) since the mesothelium is only sensitive to long fibres. Other studies have reported that intratracheal instillation, pharyngeal aspiration, or inhalation of short SWCNTs induce inflammatory and granuloma responses in the lungs of mice [31-34].

The fourth type of CNT investigated here, CNT_LONG1_, showed initial sensitivity to incubation in Gambles solution at least to 3 weeks (but possibly shorter as 3 weeks was the first interrogation time), and thereafter the product of the initial degradation appeared to be stable with regards to mass. The profile of the time-dependent dissolution of the CNT_LONG1 _sample is difficult to explain. It is possible that the rapid loss of 30% mass over the first three weeks of incubation could reflect the loss of the small, curled-up CNTs that decorated the longer fibres. However, inspection of electron microscopy images of the CNT_LONG1 _after 0 weeks or 10 weeks incubation did not reveal any obvious difference in the proportion of these decorating CNTs, suggesting that this possibility, if it occurred, was unlikely to have accounted for the total loss of mass observed. In addition, the percentage of very long fibres in this sample progressively decreased from 0 to 3 to 10 weeks, while the percentage of shorter fibres in the sample increased. These changes in morphology were accompanied by a mitigation of the strong inflammatory and fibrotic response induced by fibres incubated for 0 weeks. The inflammatory response from 0 weeks CNT_LONG1 _samples injected into the peritoneal cavities of mice is similar to that observed for LFA and 0 weeks LFC, indicating that pristine CNTs, when present as discrete long fibres, may show a pathogenicity equivalent to some forms of long fibre asbestos when compared on a mass basis, as has been observed previously [18]. However it should be noted that, because they are lighter, a much-greater *number *of CNT fibres would be present per unit weight compared with the heavier asbestos fibres.

It could be argued that because we injected a presumed dose of 50 μg based on the assumption of zero loss of mass, whereas the sample had actually shown a 30% loss of mass, the loss of pathogenicity observed for CNT_LONG1 _10 weeks could be due to injecting smaller actual doses. However, for a 30% loss of mass we saw a substantial loss of toxicity in the CNT_LONG1 _10 weeks compared to 0 weeks samples. In contrast, the LFA 10 weeks sample showed an apparent 25% loss of mass, and was also injected as a presumed dose of 50 μg, but no loss of toxicity was observed. TEM data also indicate fibre shortening in the CNT_LONG1 _and LFC samples over time which was not observed for LFA; therefore we speculate that the observed loss of toxicity with incubation in Gambles solution for both CNT_LONG1 _and LFC can be attributed at least in part to fibre shortening, consistent with a previous study that pointed to a reduced inflammogenic potential of degraded SWCNTs [14].

Donaldson *et al *[35] have proposed that, similarly to the fate of fibres reaching the parietal pleura, at the peritoneal face of the diaphragm following intraperitoneal injection, particles and short fibres may be cleared through stomata whilst long fibres that cannot negotiate the stomata are retained here, leading to inflammation and fibrosis. Under this model, the inflammatory response induced at 24 h by CNT_LONG1 _with 0 weeks incubation in Gambles solution would indicate that the long fibres of this sample are retained at the diaphragmatic stomata, and are still there at 7 d as evidenced by the development of fibrotic plaques and the continued inflammatory response. The much reduced inflammatory responses at both 24 h and 7 d induced by the CNT_LONG1 _sample incubated for 10 weeks in Gambles solution is consistent with the shorter fibres in this sample being cleared, with fewer being retained at the diaphragmatic stomata.

A question remains over the kind of chemical reaction that caused the loss of mass and fibre shortening in the CNT_LONG1 _samples that was absent in the other CNT samples. It has been suggested that synthesis defects (such as point defects, or 5-membered or 7-membered carbon rings in the sidewalls causing strain), and/or removal of impurities during or after CNT synthesis that can also introduce defects into the fibres, could act as points of weakness for mechanical or chemical attack and result in breakdown of CNTs [9]. Three recent studies investigating the biodegradation of short SWCNTs when incubated in oxidizing environments [14-16] have shown that the biodegradable SWCNTs carried surface carboxylic acid groups. One of the studies showed that only carboxylated SWCNTs were subject to biodegradation whereas pristine SWCNTs or those with other kinds of surface functionalizations were not [15]. Thermogravimetric analysis in another of these studies [16] also showed a loss of mass in carboxylated SWCNTs alongside biodegradation comparable to the loss of mass we saw in our CNT_LONG1 _sample. These studies did not address the biodegradation of MWCNTs. When we analysed our CNT samples by X-ray photoelectron spectroscopy (XPS), we found that the most oxygenated sample was CNT_SW_, which had no mass loss. However, the durable CNT_TANG2 _and the degradable CNT_LONG1 _had oxygen levels similar to each other, but lower than observed for CNT_SW_. We did not measure if the surfaces of our samples were oxidized, but oxidative attack on MWCNTs may result in successive layer removal leading to thinner tubes [36], which we did not see. Future studies are required to investigate the mechanism(s) driving mass loss and fibre-shortening leading to the reduced pathogenicity observed here for CNT_LONG1_, and to determine if the mechanisms are applicable to other MWCNTs.

## Conclusions

In summary, we have found that three of the four types of CNTs tested here showed close to 100% durability after incubation for 24 weeks in a simulated biological fluid, while a fourth lost mass, accompanied by a reduction in the proportion of long fibres in the sample. It is important to note that three types of CNTs assessed here were durable and therefore the fibre shortening and loss of mass seen in the fourth sample (CNT_LONG1_) cannot be generalised across all types of CNTs. Subsequent testing for inflammogenic potential using a fibre-specific assay revealed that an adverse response *in vivo *was dependent on both the durability and the presence of discrete, long CNTs or fibre-shaped agglomerates of CNTs in the sample: the durable but tightly agglomerated bundles of short CNT_SW _elicited a minimal response in mice, while the pristine, discrete, long, thin fibres of CNT_LONG1 _induced an asbestos-like response, which was mitigated after chemical incubation reduced the proportion of fibres longer than 15 μm. These findings add to growing evidence that biodurability and pathogenicity are not consistent across all types of CNTs. However, given the substantial response induced in mice by the pristine fibres of CNT_LONG1_, and the fact that the other three types of CNTs tested here were durable, we would suggest that CNTs that are potentially of pathogenic fibre dimensions should be treated with a very high level of caution in the workplace to avoid inhalation, as it is expected that the majority of CNTs may be biopersistent. Clearly, though, if a CNT can be designed and manufactured with safety in mind without compromising its intended application, for example with some kind of surface defect that makes it vulnerable to chemical attack and degradation in biological systems, or with a propensity to form clump-like agglomerates that can be cleared, then there is the potential for biological hazards to be minimised.

## Methods

### Characterisation of test samples

Seven fibrous samples were tested for their durability in Gambles solution: four types of CNTs and three control fibres. Two of the MWCNTs (CNT_LONG1 _and CNT_TANG2_) and the SWCNT (CNT_SW_) were commercial samples obtained from Mitsui & Co., NanoLab and Sigma-Aldrich, respectively. The third type of MWCNT tested (CNT_SPIN_) was prepared using a proprietary method and supplied by the CSIRO (Australia). For controls, we assessed two types of asbestos fibres [amosite (LFA) and chrysotile (LFC)] and one type of glass wool fibre (X607). Two of the CNT samples used in this study (CNT_LONG1 _and CNT_TANG2_) have been described elsewhere [18]. However, these samples were characterised again in this study to maintain consistency across samples.

For quantification of contaminating metals, approximately 0.2-2.7 mg/ml of each sample was weighed into an acid-cleaned digestion tube containing 0.2% (v/v) HNO_3_. The samples were mixed and digested at room temperature for 15 min after which the 0.2% HNO_3 _leach solution was filtered through acid-cleaned 0.45 μm filter cartridges and analysed by matrix-matched standards using the techniques of inductively-coupled plasma mass spectrometry (ICP-MS, Agilent 7500 CE) or inductively-coupled plasma atomic emission spectroscopy (ICP-AES, Varian 730-ES).

For measurement of endotoxin levels, 1 mg/ml each sample was vortexed for 1 min in limulus amebocyte lysate (LAL) endotoxin-free water and incubated for 1 h at 37°C. Samples were then centrifuged and endotoxin levels in the supernatant were determined in triplicate using the QLC-1000 Chromogenic LAL kit (Lonza, Australia) following manufacturer's instructions. A previous trial had shown that centrifugation did not artificially lower endotoxin levels in the supernatant. An aliquot of each supernatant was also spiked with a known amount of endotoxin and measured alongside unspiked samples to confirm the absence of assay inhibition.

Samples were assessed for their potential to generate free radicals by electron paramagnetic resonance (EPR). TEMPONE-H (Enzo Life Sciences) was used as a spin trap to quantify peroxynitrite and superoxide radical formation. Samples were prepared by diluting the filtered test samples that had been resuspended for injection to 0.01 mg/ml in saline. TEMPONE-H (1 μl of 100 mM stock solution) was added to 99 μl of the diluted test sample to obtain a final concentration of 1 mM TEMPONE-H. The samples were incubated at 37°C for 60 min after which the levels of oxidised TEMPONE-H were quantified by EPR. Undiluted test samples at a presumed mass of 0.5 mg/ml were also assessed. Pyrogallal in Hanks Buffered Solution was a positive control for the TEMPONE-H reaction. A negative control for the 0.01 mg/ml samples was prepared by adding 2 μl 0.05% BSA:saline to 97 μl saline. A negative control for the undiluted test samples was prepared by using 99 μl 0.05% BSA:saline.

XPS analysis was performed using an AXIS Ultra DLD spectrometer (Kratos Analytical Inc., Manchester, UK) with a monochromated Al K_α _source at a power of 150 W, a hemispherical analyser operating in the fixed analyser transmission mode and the standard aperture (0.3 mm × 0.7 mm slot). The total pressure in the main vacuum chamber during analysis was typically less than 5 × 10^-8 ^mbar.

Each specimen was analysed at an emission angle of 0°as measured from the surface normal. Since the actual emission angle is ill-defined in the case of particles (ranging from 0° to 90° with respect to the surface normal) the sampling depth may range from 0 nm to approximately 10 nm. All elements present were identified from survey spectra (acquired at a pass energy of 160 eV). The atomic concentrations of the detected elements were calculated using integral peak intensities and the sensitivity factors supplied by the manufacturer. The accuracy associated with quantitative XPS is ca. 10% - 15%. Precision (i.e. reproducibility) depends on the signal/noise ratio but is usually much better than 5%. The latter is relevant when comparing similar samples.

Full characteristics of the test samples are given in Table 5.

**Table 5:** Physicochemical properties of control fibres and CNT samples

Sample Name	Description	Diameter as supplied by manufacturer (nm, mean(SE)	Length as supplied by manufacturer μ(m)	Endotoxin^1 ^(pg ml^-1^)	Surface groups (atomic ratio (X/C))	Soluble metals (μg g^-1^)	Free radical generation	Morphology (SEM, TEM, Light Microscopy)
**X607**	Glass fibres	NA	NA	ND	N/A	Li-0.41; Be-0.02; Al-350^2^; V-1.07; Cr-4.05; Mn-29.6; Fe-314^2^; Co-0.08; Ni-2.62; Cu-0.72; Zn-7.09; As-0.12; Sr-48.4; Mo-0.25; Ag- < 0.01; Cd-0.02; Sb-0.01; Pb-0.43; U-0.03	None	Dispersed rod-like glass fibres.

**LFA**	Amosite asbestos	NA	NA	ND	N/A	Li-0.15; Be-0.15; Al-463; V-1.62; Cr-4.45; Mn-622; Fe-~3200^3^; Co-0.45; Ni-2.67; Cu-2.96; Zn-2.9; As-0.07; Sr-29.2; Mo-0.45; Ag- < 0.03; Cd-0.02; Sb- < 0.04; Pb-0.79; U-0.04	None	Dispersed rod-like amphibole asbestos.

**LFC**	Chrysotile asbestos	NA	NA	ND	N/A	Li- < 0.14; Be-0.01; Al-137; V-1.29; Cr-32.3; Mn-83.1; Fe-1220; Co-5.54; Ni-140; Cu-0.77; Zn-10.9; As- < 0.11; Sr-2.26; Mo-0.08; Ag- < 0.08; Cd-0.04; Sb- < 0.09; Pb-0.46; U-0.02	None	Dispersed fibrous-looking chrysotile asbestos.

**CNT_SW_**	Single-walled	1-2	0.5-2	ND	C-1.0000; O-0.0105; Si-0.0004; Fe-ND; Cl-0.0005; S-0.0002; Co-0.0007; Ni-0.0004	Li- < 0.04; Be- < 0.003; Al-6.2; V-0.34; Cr-2.01; Mn-15.7; Fe-185; Co-442; Ni-47.4; Cu-1.13; Zn-2.9; As-0.23; Sr-2.72; Mo-144; Ag-0.03; Cd-0.1; Sb- < 0.03; Pb-0.98; U-0.04	None	Bundles of tightly agglomerated SWNTs in which individual NTs cannot be seen

**CNT_SPIN_**	Multi-walled	8-10	200-300	ND	C-1.0000; O-0.0013; Si-0.0007; Fe-0.007; Cl-ND; S-ND; Co-0.0002; Ni-0.0002	Li- < 0.02; Be- < 0.001; Al-0.3; V-0.01; Cr-0.07; Mn-0.02; Fe-50.1; Co- < 0.003; Ni-0.46; Cu-0.16; Zn-0.95; As-0.12; Sr-48.4; Mo-0.25; Ag- < 0.01; Cd-0.02; Sb-0.01; Pb-0.43; U-0.001	None	Agglomerated sheets of very long fibres with a hair-like appearance.

**CNT_LONG1_**	Multi-walled	40-50	Mean 13	ND	C-1.0000; O-0.0046; Si-0.0012; Fe-ND; Cl-0.0001; S-0.0002; Co-0.0001; Ni-0.0002	Li- < 0.09; Be- < 0.007; Al-0.9; V-0.01; Cr-0.15; Mn-0.09; Fe-15.6; Co- < 0.01; Ni-0.2; Cu-0.06; Zn-2.5; As- < 0.07; Sr-0.84; Mo-0.01; Ag- < 0.05; Cd- < 0.02; Sb- < 0.06; Pb-0.03; U-0.01	None	Bundled or individual MWNTs of variable length, many in the 10 20(m range or longer. Many very short fibres often decorate the longer fibres.

**CNT_TANG2_**	Multi-walled	15 ± 5	5-20	ND	C-1.0000; O-0.0038; Si-0.0009; Fe-ND; Cl-ND; S-0.0005; Co-0.0007; Ni-0.0003	Li- < 0.08; Be- < 0.006; Al-41.6; V- < 0.01; Cr-0.03; Mn-0.05; Fe-606; Co-0.04; Ni-0.44; Cu-1.07; Zn-9.5; As- < 0.07; Sr-0.3; Mo-655; Ag- < 0.05; Cd-0.04; Sb- < 0.06; Pb-0.26; U- < 0.006	None	Bundles of intermediate-length MWNTs. Often stellate in form with longer fibres protruding from the central tangled agglomerate, a large proportion of which are in respirable size range < 5 (m.

Note: Lengths and widths as determined by authors can be found in Table 3

NA: Not available

ND: Not detected

N/A: Not applicable

^1^Endotoxin detection limit < 10 pg ml-1

^2^Analysed by ICP-AES

^3^Value over range, and is an estimate only

### *In vitro *assessment of biodurability

Gambles solution was prepared the day before addition to the samples (per litre: 7.12 g NaCl; 1.95 g NaHCO_3_; 0.029 g CaCl_2_.2H_2_O; 0.148 g Na_2_HPO_4_; 0.079 g Na_2_SO_4_; 0.212 g MgCl_2_.6H_2_O; 0.118 g Glycine; 0.152 g Trisodium citrate.2H_2_O; 0.18 g Disodium tartrate.2H_2_O; 0.172 g Sodium pyruvate; 167 μl lactic acid). Formaldehyde (2 ml/L; 37% in formalin) was added to prevent microbial growth. The pH was adjusted by HCl to 4.5 and then readjusted the next day prior to addition to the samples.

For each time-point of interest, four replicates of each test sample (each weighing 1.00-3.00 mg) were weighed (Ohaus AP2500) into 7 ml plastic flat-bottomed Bijou tubes. An appropriate amount of Gambles solution was added to each sample to give a final concentration of 0.5 mg sample/ml. Samples were sonicated (Fisherbrand ultrasonicating water bath, ultrasonic frequency: 40 kHz) until the CNTs or fibres were visually judged to have dispersed as well as possible. Consequently, the X607, LFA and LFC samples were sonicated for 20 min and the CNT samples were sonicated for 1 h. All samples were then incubated with shaking for up to 24 weeks at 37°C with the Gambles solution refreshed every 3 weeks. 0 weeks samples were treated identically to incubated samples with the exception that after mixing with Gambles solution they were immediately filtered and dried, without sonication.

The four replicates of each sample were removed from incubation at 0 weeks, 3 weeks, 6 weeks, 10 weeks and 24 weeks and filtered and washed with double-distilled water (ddH_2_O) onto pre-weighed PVC filter papers (5.0 μm pore size, 25 mm diameter, SKC Inc). Blank Gambles solution was also washed through five filter papers to control for mass not attributable to the recovered CNTs or fibres. The filtered samples were left to dry protected from dust at room temperature for five days, and then weighed to 0.001 mg (Sartorius MC5-OCE), and percent recoveries relative to recovery at 0 weeks were calculated.

Statistical significance of loss of mass across the time points for each sample type was assessed by one-way analysis of variance (ANOVA) with Tukey's Multiple Comparison post test.

For morphological assessment by SEM, samples of each CNT or fibre that had been incubated in Gambles solution for 0 weeks or 10 weeks were filtered on Whatman PC filter membranes (0.2 μm pore size, 25 mm diameter) and viewed by SEM (Hitachi S-2600N). EDX (Inca System, Oxford Instruments) linked to the SEM was used to confirm that the major elements present in the samples being viewed were consistent with what was expected.

For measurement by TEM, samples of each CNT or fibre that had been incubated in Gambles solution for 0 weeks or 10 weeks were filtered on PVC filter papers (5.0 μm pore size, 25 mm diameter, SKC Inc.). To prepare the samples for TEM, small fragments of filtered material were removed from filter paper with forceps and suspended in 50-100 μl ethanol by gentle sonication in a water bath for up to 3 h, with samples of the same type always sonicated for equivalent periods of time. Addition of 1% triton or sonication for longer times did not improve the degree of dispersion. Aliquots of each dispersion (5 μl) containing small fragments of material (visible to the naked eye) were pipetted onto carbon-coated 100 mesh copper grids. Grids were examined in a Tecnai 12 TEM (FEI, Eindhoven, Netherlands) operating at 120 kV at a variety of different magnifications. Images were recorded using a MegaView III CCD camera (Olympus) and AnalySiS software. Measurements were made using Image J (NIH) calibrated via the embedded scale bar.

The technical difficulties in measuring the lengths and diameters of CNTs within bundles should be noted here, and therefore the measurements included in Table 3 should be regarded only as trends rather than absolute values. For the measurements reported here, a number of images for each sample were examined and all fibres in a given image that had a discernible length or width were measured for that aspect. When a straight fibre passed over a grid line and did not emerge from the other side, the mid-point of that grid line was measured as the end of that fibre. If two or more CNTs overlapped with one another such that the lengths and/or diameters of individual fibres could not be determined, those CNTs were excluded from measurement. As we were interested in time-dependent alterations in the proportions of long to short fibres, we included fibres with lengths > 5 μm for representative purposes, although these measurement rules depart from the World Health Organisation's guidelines.

To increase confidence in our measurements, two sets of measurements were performed by two individual operators and an average of 100 measurements was made per sample, although the number of measurements for samples in which individual fibres were difficult to identify were considerably lower (e.g. only 23 measurements were possible for determining the lengths of CNT_SW_).

### *In vitro *determination of the impact of sonication on CNT_LONG1 _mass loss and fibre changes

Quadruplicate CNT_LONG1 _samples were weighed as described, then either sonicated in Gambles solution for 1 h under conditions identical to the main experiment and filtered, dried and weighed, or washed in Gambles solution and immediately filtered, dried and weighed, replicating the conditions by which we produced our original 0 weeks samples. Sample recoveries and fibre lengths were measured as described above.

### *In vivo *assessment of inflammogenic potential

Samples of X607, LFA, LFC, CNT_SW _and CNT_LONG1 _that had been filtered after 0 weeks or 10 weeks incubation were used to investigate the impact of incubation in Gambles solution on inflammogenic potential *in vivo *in mice. CNT_TANG2 _was not assessed as it had previously been shown not to elicit an inflammatory response in mice [18]. CNT_SPIN _was not assessed due to sample characteristics that made it impossible to obtain a good dispersion in 0.5% BSA:saline, and therefore we could not be confident of injecting a known mass for *in vivo *evaluation.

Four female C57Bl/6 mice aged 8 weeks were used for each sample type at each selected time-point, plus three control mice that were injected with vehicle only. Mice were housed at the University of Edinburgh Biological Research Facility under standard housing conditions of 12 h light/dark cycles, and food and water was available *ad libitum*. All procedures were conducted in accordance with Edinburgh University guidelines.

The samples (residues after filtering, washing and drying) were resuspended with sonication in 0.5% BSA (1 h bath sonication (Fisherbrand ultrasonicating water bath) ultrasonic frequency: 40 kHz) followed by 10 sec probe sonication (Bandelin Electronics Status US 70, Berlin, Germany: 60% power using pulsing) in sterile saline at their presumed original mass of 0.5 mg/ml. Based on this presumed 100% recovery of original mass, 50 μg each sample, or vehicle only, was injected into the mouse peritoneal cavity. After 24 h or 7 d the mice were sacrificed by asphyxiation in 100% CO_2 _and the peritoneal cavity of each mouse was washed (lavaged) three times with 2 ml sterile saline using a 21 gauge needle. The first wash was stored in a chilled 1.5 ml Eppendorf tube, and the second and third washes were combined in a conical 15 ml falcon tube, both on ice.

Following sacrifice and lavage at the 7 d time-point only, the peritoneal cavity was exposed via lateral incisions in the abdominal wall extending to the vertebral column, which was severed below the diaphragm. The ribcage around the diaphragm was then cut from each mouse, taking care not to tear the diaphragm, rinsed by gentle immersion in ice-cold sterile saline and stored in 'methacarn' fixative (60% methanol, 30% chloroform, 10% acetic acid) at room temperature for approximately five days. The diaphragm was then carefully excised from the surrounding ribcage and stored in 70% ethanol until processed for histology.

To pellet cells obtained by lavage, samples in the 1.5 ml tubes were centrifuged at 2000 rpm for 5 min at room temperature and the combined washes in the 15 ml tubes were centrifuged at 123 g for 5 min at room temperature. From the first wash a 1 ml aliquot of supernatant was transferred to a fresh 1.5 ml Eppendorf tube and stored at -20°C for quantification of Interleukin-6 (IL-6). A further 200 μl from the first wash was combined in a fresh 1.5 ml Eppendorf tube with 400 μμl supernatant from the second and third washes and stored at 4°C for quantification of lactate dehydrogenase (LDH) and total protein levels.

To prepare slides for total and differential cells counts, the remaining supernatants were discarded and the cell pellets from each of the second and third washes were resuspended in 500 μl 0.01% BSA:saline, and then combined with the pellet from the first wash, which was completely resuspended by gentle pipetting. An aliquot (10 μl) was diluted 1:10 in sterile saline and total cell numbers were counted using a NucleoCounter (ChemoMetec, A/S, Allerød, Denmark) following standard protocol. Briefly, to the 100 μl containing 10-fold diluted samples was added 100 μl lysis buffer (ChemoMetic Reagent A) followed by 100 μl stabilisation buffer (ChemoMetic Reagent B). The lysed solution was drawn up into a NucleoCounter and the diluted cell count obtained.

After total numbers for each sample had been calculated (diluted cell count X10), samples were prepared for differential cell counting. Glass slides (Thermoscientific) were labelled and then placed in a cytospin slide cassette with filter cards (ThermoShandon) and placed in a cytospin centrifuge (Cytospin 4, ThermoShandon). BSA (300 μl 0.01% in saline) was added to the cassette and the cell suspension was added at an appropriate volume to obtain ~40,000 cells/slide. The cassettes were centrifuged at 300 rpm for 3 min at room temperature after which the slides were removed and allowed to dry at room temperature. Once dried, the slides were fixed in 100% methanol, and then stained with eosin (DiffQuickI, Dade Behring) followed by hematoxylin (Quick-Diff Blue, Reastain). Differential cell counting was performed using light microscopy.

The bicinchinonic acid (BCA) protein assay (Sigma Aldrich) was used to measure total protein concentration of the peritoneal lavage fluid. The test reagent (1 part copper (II) sulphate solution (4% w/v) to 50 parts BCA) was added to the standards and lavage samples, which were then incubated at 37°C for 30 min. The absorbance of solutions in plates was read at 570 nm using a Synergy HT microplate reader (BioTek Instruments). The protein concentration of each sample was established by interpolation from a BSA standard curve (0-1000 μg/ml).

Concentrations of IL-6 in the peritoneal lavage samples were determined by ELISA (IL-6 Duoset, R&D Systems). The samples and standards were added to 96-well plates pre-coated with IL-6 capture antibody and incubated at room temperature for 2 h. Each well was thoroughly washed with 0.05% Tween 20/PBS before incubation with IL-6 detection antibody at room temperature for 2 h. Wells were washed again followed by 20 min incubation with Streptavidin-HRP conjugate. The wash step was repeated before the addition of substrate solution (3,3',5,5'-tetramethylbenzidine, Sigma) to each well. Plates were incubated in the dark at room temperature until the colour had developed sufficiently, after which the reaction was stopped by the addition of 50 μl 2 N H_2_SO_4 _per well. Absorbance at 450 nm was read. The IL-6 concentration in each sample was established by comparison with an IL-6 standard curve (0-100 pg/ml).

Levels of LDH in the peritoneal lavage fluid were assessed using the LDH detection kit (Roche Applied Science). Following addition of the test reagent (diaphorase/NAD^+ ^catalyst mixture diluted in iodotetrazolium chloride (INT) and sodium lactate dye solution), samples were incubated in darkness at room temperature for 30 min after which absorbance was read at 490 nm.

To detect the presence of fibrotic plaques in the excised diaphrams, and measure their sizes, 4 μm-thick diaphragm sections were stained with hematoxylin and eosin, and serial images were taken using QCapture Pro software (Media Cybernetics Inc., MD, USA). The images were realigned using Photoshop Elements 4CS3 (Adobe Systems Inc.) and the total length of each diaphragm along the basement membrane was measured using calibrated Image-Pro Plus software (Media Cybernetics Inc., MD, USA). The area of each detected fibrotic plaque was measured, excluding areas of adherent tissue such as liver, connective tissue or lymphatic tissue. The area of fibrotic plaque on each diaphragm (in mm^2^) was expressed per unit length of diaphragm (in mm) to give fibrotic plaque area per unit diaphragm length (mm^2^/mm). Average results were calculated from four separate animals for each treatment and statistical significance was assessed by one-way ANOVA with Tukey's Multiple Comparison post test.

### Statistics

All data are expressed as the mean ± the standard error of the mean (SE). Statistical tests were performed using GraphPad Prism 5.01 (GraphPad Software, San Diego, USA). Comparisons between two groups were analysed using unpaired t-tests, and multiple comparisons were analysed using one-way ANOVA with Tukey's Multiple Comparison post test. For all tests, values of *p *< 0.05 were considered statistically significant.

## Competing interests

The authors declare that they have no competing interests.

## Authors' contributions

MO drafted the manuscript, and contributed to experimental work and data analysis. CP, FM and LW contributed to experimental work, data analysis, and manuscript preparation. SC contributed to experimental work. SCH prepared and supplied the CNT_SPIN _sample and contributed to experimental work. MJM, HM, RA and KD initiated the study, oversaw all experimental work, contributed to data analysis, and contributed to manuscript preparation. All authors read and approved the final manuscript.
